# Impact of Urbanisation Intensity on Bird Diversity in River Wetlands around Chaohu Lake, China

**DOI:** 10.3390/ani12040473

**Published:** 2022-02-14

**Authors:** Qingru Xu, Lizhi Zhou, Shanshan Xia, Jian Zhou

**Affiliations:** 1 School of Resources and Environmental Engineering, Anhui University, Hefei 230601, China; xuqr1028@163.com (Q.X.); xss051533@163.com (S.X.); 18405512357@163.com (J.Z.); 2 Anhui Province Key Laboratory of Wetland Ecosystem Protection and Restoration, Anhui University, Hefei 230601, China

**Keywords:** wetland protection, bird community, redundancy analysis, habitat loss

## Abstract

**Simple Summary:**

The continuous intensification of urbanisation has led to severe degradation and loss of bird habitats, directly affecting the diversity of birds. In this study, we focused on seven representative river wetlands around Chaohu Lake (China) to analyse the impact of urbanisation on bird diversity. The species richness at sections of the lake entrance was higher than in the middle sections of the river, and the Shannon–Wiener index during autumn was higher than that during winter. Urbanisation was an important driving factor that changed land use types near rivers and the structure of bird communities. The response model of river ecological indicators to the intensity of urbanisation shows a negative exponential correlation between the waterbird diversity index and the urbanisation intensity. Our research is of great significance for future urban landscape planning and bird community diversity protection.

**Abstract:**

Urbanisation is known to result in ‘urban stream syndrome’, which poses a huge threat to the river health. Birds, which are an important part of the river ecosystem, are sensitive to environmental changes in the basin. The ratio of the impervious surface area is a macroscopic indicator of urbanisation intensity in river basins. In this study, we combined the results of a year-round field survey of seven river wetlands around Chaohu Lake (China) with satellite remote sensing image data from the same period. The species richness at sections of the lake entrance was higher than in the middle sections of the river, and the Shannon–Wiener index during autumn was higher than that during winter. The waterbird diversity index declined exponentially with increases in the intensity of urbanisation. The changes in the land use patterns around river wetlands associated with urbanisation resulted in the loss of food resources and habitats. Therefore, the intensity of urbanisation was an important driving factor that leads to changes in the bird community structure of river wetlands, so it had a significant impact on the diversity of river wetland birds in all four seasons combined with a variety of influencing factors. Our research could be a guide for urban landscape planning and bird diversity protection. For example, the results suggested that it is necessary to identify river wetlands as an important part of the urban ecosystem, reduced building area, increased vegetation coverage, and retained slope protection and river beach land.

## 1. Introduction

Globally, the continuous intensification of industrialisation and urbanisation has led to a significant reduction in wetlands and land cover due to newly constructed lands. Although cities account for less than 3% of the land area on Earth, the loss of native habitats resulting from urbanisation has become an important cause of decreasing biodiversity [1]. Birds, as a large and widely distributed group of animals, are sensitive to changes in habitats and human disturbance. They have high mobility and habitat selectivity [2] and are therefore often selected as biological indicators of habitat and ecosystem changes [3].

River wetlands not only are important in terms of ecology, landscape, and social benefits but also are a relatively complete habitat structure that includes rivers, beaches, rocks, and grassland; these areas are crucial breeding sites for many species [4]. Especially in cities, river wetlands provide places for birds and other wildlife to forage and rest [5]. However, they have been buried, cut, and hardened; thus, the animal diversity has significantly declined in recent years. These issues raise awareness of the urgency in and result in actions of preserving the remaining river wetlands worldwide [6]. Bird monitoring data from river wetlands have great significance for protecting biodiversity and for monitoring ecosystem health. For example, the index of bird community integrity (IWCI) can be used to monitor the integrity of river ecosystems and to assess the impact of the composition of a bird community on the food web in a lagoon [7,8].

Thus far, several studies on different temporal and spatial scales have helped determine the key factors that affect the diversity of river wetland bird communities. Water is an essential component, acting as an artery of the river ecosystem [9]. High water quality is conducive to the survival of aquatic organisms, enriching the food resources of birds and increasing the diversity of bird [10,11]. Hoyer et al. showed that the abundance of waterbird species richness was positively correlated with the size of the water body [12]. Additionally, the nutritional status of the lake had a significant positive impact on the diversity of waterbirds [13,14,15]. The encroachments on the river due to built-up areas, bridges, etc. led to the disappearance of natural habitats for birds and other biodiversity [16]. The diversity of birds dropped with higher road and building surface coverage [17]. A gradient study of the Salt River (which passes through Phoenix in AZ, USA) revealed that land-use types near the river were clearly related to the composition of wintering waterbird species [18]. Researchers found higher bird diversity in the river habitats of rural areas in Florida than in urban areas during the summer, and the housing density on adjacent land decreased the size of bird communities in river wetlands [19]. Cities that were built along rivers occupied bird habitats, thus reducing its diversity [20]. Several studies implied that using gradients to describe the distribution of landscape configurations in urban areas was ideal for studying birds adapting to land cover changes [1,4,5].

We studied the diversity of birds under different river wetlands and different urbanisation intensities in this paper. The main aims of the study were to determine (a) the key environmental factor that affected the bird community diversity (species richness, Shannon-Wiener and Pielou indices) in different seasons and (b) the intensity of urbanisation of river wetlands around Chaohu Lake (China) that affected the diversity of the bird community, and their relationship. We hypothesised that urbanisation intensity, river section, and season all affected bird diversity in river wetlands. We predicted that habitat loss would be the main driver of bird urbanisation and that there was a negative correlation between the intensity of urbanisation and the diversity of birds. This is because the intensity of urbanisation plays a major role in determining the structure of bird community, especially for waterbirds.

## 2. Materials and Methods

### 2.1. Overview of the Research Areas

**Figure 1:**
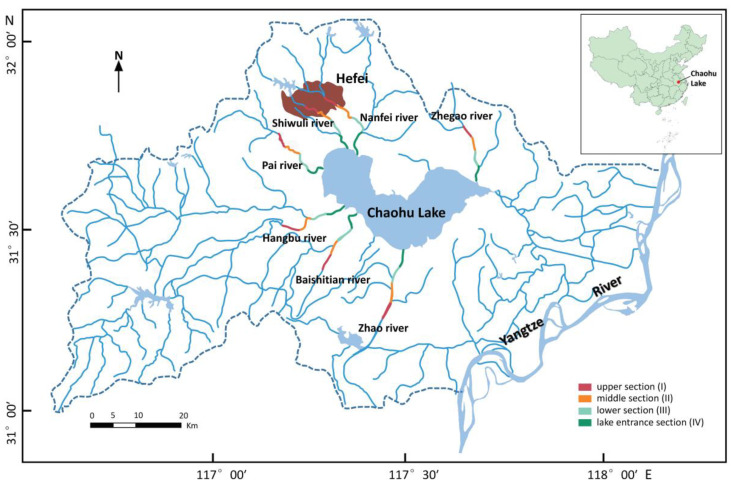
Study area with the seven rivers entering Chaohu Lake, China (the brown area is Hefei, the capital of China’s Anhui province).

Chaohu Lake (117°16′46′′–117°51′51′′ E, 31°25′28′′–31°43′28′′ N) is one of the five major freshwater lakes in China, located in Hefei, Anhui Province (Figure 1) [21]. The permanent population of Hefei is as high as 9.369 million, of which 7.709 million live in cities, which takes up 82.3% of the total population (data downloaded from http://tjj.hefei.gov.cn/tjyw/tjgb/14735553.html, accessed on 27 December 2021). The basin around Chaohu Lake includes diverse types of wetlands; among them, river wetlands comprise a major part of this area [22,23]. The river network is densely distributed, with an asymmetrical water system that flows into the lake from the south, west, and north [24]. This terrain in the area is undulating and rough, with abundant mountain rivers [25]. The largest rivers that enter the lake include the Hangbu, Baishitian, Nanfei, Pai, Zhegao, and Zhao Rivers, with the first three rivers accounting for 70% of the area of Chaohu Lake basin and the Shiwuli River, Nanfei River, and Pai River in Hefei being the three most polluted rivers, in that order [26,27,28,29]. Therefore, we selected seven representative river wetlands: Zhao River Wetland, Zhegao River Wetland, Hangbu River Wetland, Baishitian River Wetland, Nanfei River Wetland, Shiwuli River Wetland, and Pai River Wetland. In this study, we selected a 20 km river area away from the entrance of the Chaohu Lake and a buffer zone extending 200 m outwards on the left and right sides of the water body boundary as the study area, considering the physical form of the rivers and the topography. The area comprising the river section was evenly divided into the upper (I), middle (II), lower (III), and lake entrance (IV) sections [30,31,32]: section I was denoted the river section group I, section II of each area was denoted the river section group II, section III was denoted the river section group III, and section IV of each area was denoted the river section group IV [33,34,35]. We set the distance between the different river sections to 2 km to study the impact of different river sections being inhabited on birds. This study could reveal the current bird distribution and diversity in the different river sections.

### 2.2. Data Collection and Analysis

#### 2.2.1. Bird Surveys

The survey was conducted from June 2020 to May 2021. The year was divided into summer (June to August), autumn (September and October), winter (November, December, and January to March of the following year), and spring (April and May), according to the annual life cycle of migratory birds [36]. We followed the route survey method described by Xu et al. [3] to count birds for 12 months in seven river wetlands (Zhao River, Zhegao River, Hangbu River, Baishitian River, Nanfei River, Shiwuli River, and Pai River) within the Chaohu Basin of Anhui Province [37]. Three sampling routes measuring 1 km were arranged along each river section, with an interval of 0.5 km between each sample route. All sample routes were close and parallel to the riparian. For each survey, two professionally trained field surveyors conducted bird surveys at a speed of 2 km/h. Each survey was completed by the same two investigators, one of whom recorded and the other monitored to eliminate subjective errors. We used binoculars and monocular telescopes to observe the birds at a fixed angle and counted the birds above the river and the birds within 50 m of the river bank (only birds in or leaving the sample route were recorded, and birds flying over the sample route were not included) [37]. We used a combination of precise counting and estimation; birds in small groups were counted directly, and the ‘group counting method’ was adopted to count the number of birds in larger groups [36]. For example, the bird community was divided into groups of different sizes, with 10, 50, or 100 individuals comprising the larger groups. The total number of birds and the percentage of each species were calculated based on the number of field observations. Surveys were conducted on fixed dates once each month, when possible. Additionally, the survey was postponed if the weather was poor. Surveys were conducted every ten days on fixed routes at 3 h after sunrise and 3 h before sunset, and in sunny weather without strong wind (speed > 30 km/h) only. The investigators recorded the Global Positioning System (GPS, eTrex30, Garmin, China) location of every observation point using a handheld GPS.

#### 2.2.2. Measurement of Environmental Factors

We determined 11 environmental factors and divided them into habitat and landscape factors based on field investigations and existing research. Five types of habitat factors were identified: potential of hydrogen (pH), dissolved oxygen (DO), water temperature (WT), transparency (SD), and the degree of human disturbance (disturb). There were six types of landscape factors: the ratio of the impervious surface area (PLAND_i), the ratio of the water area (PLAND_w), the ratio of the forest area (PLAND_f), habitat heterogeneity (SHDI), the distance from the Hefei centre (distance), and the width of each river section (width).

The habitat factors pH, DO, WT, and SD were used to evaluate the quality of river water. The pH, DO, and WT habitat factors were measured with a portable water quality analyser (Hach HQ40d, USA) in the field. We put the instrument probe into the river water, then carried out the test and collected the result data. The basic standard integrated parameter of surface water layer transparency (SD) was the depth at which the white disk (Secchi disk) disappeared from the surface observer’s view [38]. The degree of human disturbance was an estimated value. The level of human interference was divided into five levels according to human activity and the presence of machinery in the research area: Level 1, no interference, unmanned, no construction noise, and no motor vehicles or ships present; Level 2, low interference, 1–3 people per survey, no construction noise, and no motor vehicles or ships present; Level 3, moderate interference, 3–5 people per survey, no construction noise, and no motor vehicles or ships present; Level 4, more significant interference, 5–7 people/every survey, slight construction noise, mobile motor vehicles, fishing, and some ships present; and Level 5, strong interference, many humans, significant amounts of construction noise, abundant motor vehicles, fishing, and several ships present in the area. The observer stood in place to evaluate the degree of interference, such as the flow of people, noise, vehicles, and ships, and recorded the results of the assessment. The time of each in situ observation was no less than 10 min [35]. We repeated the sampling three times for each river section at 0.5 km apart. Then, we averaged three repeated results and recorded the factor value of each section [34].

We obtained the landscape factors PLAND_i, PLAND_w, PLAND_f, and SHDI via remote sensing. Four sets of Sentinel-2 remote sensing images were downloaded (https://scihub.copernicus.eu/dhus/#/home, accessed on 24 August 2021) for the period June 2020 to May 2021 in accordance with the seasons. Additionally, the images were acquired as close as possible to the times during which the bird surveys were performed. Then, SNAP (SNAP v6.0. http://snap.stanford.edu/snap/download.html, accessed on 26 August 2021) and The Environment for Visualizing Images (ENVI v5.3. https://envi.geoscene.cn/, accessed on 27 August 2021) were used to pre-process the remote sensing images. We combined the outcomes of previous research and field investigations with the spatial distribution characteristics of the landscape in which Chaohu Lake lies [24,25,26,27,28,29] to interpret the images using supervised classification and neural networks. Then, we divided the images into six categories: the impervious surface (soil covered with impervious materials such as concrete, metal, glass, tarmac, and plastic), unused land (areas that are not suitable for agriculture or construction), forest (natural or artificial forest land with crown density > 30% and surrounded by small shrubs), water (in which surfaces are not covered by aquatic plants), cropland (areas used in the production of crops for harvest), and grassland (herbaceous plants surrounding the wetland). Tests investigating the classification accuracy indicated an accuracy higher than 90%, suggesting that the classifications were sufficient [39]. The interpretation results were cut in ArcMap (ArcMap v10.2. https://developers.arcgis.com/, accessed on 28 August 2021), then converted to tiff format, and imported into Fragstats (Fragstats v4.2. https://fragstats.software.informer.com/4.2/, accessed on 30 August 2021) to calculate the land type area (unit: km^2^) in each river section [40]. The distance and width were measured using Google Earth [41].

We collected the data of habitat factors every month at the same time as the bird survey. Additionally, we collected data on the landscape factors once per season [42,43].

#### 2.2.3. Statistical Analysis

We compared bird community diversity (the species richness, Shannon–Wiener, and Pielou indices) between rivers, seasons, and river sections using the Kruskal–Wallis test. Then, post hoc pair-wise tests were performed using the Dunn–Bonferroni test (default significance level was set at the *p* < 0.05 level).

We used a Pearson correlation analysis to assess the redundancy of environmental variables and to eliminate environmental factors with a strong correlation with other factors to avoid collinearity between environmental factors. The results showed that WT, SD, and disturb had strong correlations with other factors in summer (Figure S1a); WT, SD, disturb, DO, and SHDI had strong correlations with other factors in autumn (Figure S1b); disturb, distance, DO, and SHDI had strong correlations with other factors in winter (Figure S1c); and disturb, distance, and SHDI had strong correlations with other factors in spring (Figure S1d). These factors were therefore removed from subsequent analyses.

Redundancy analysis (RDA) is commonly used in ecology to analyse the relationship between a biological community and its environment [44]. In this study, we selected 24, 32, 29, and 24 species of birds separately for RDA during summer, autumn, winter, and spring, and the selected species had an appearance frequency of ≥20% in each river section. Then, we used the four seasons in the bird species matrix and the four seasons in the environmental factor matrix as the input for RDA and analysed the relationship between river wetland bird communities and environmental factors by season. The results were represented as a double-sequence diagram that demonstrated the relationship between species and environmental factors. Then, we made an lg (x + 1) conversion for the environmental parameters to let it tend towards a normal distribution and to eliminate the influence of extreme species richness values on the classification scores. We reduced the weight of rare species, allowing the research to focus on highly abundant species.

The urbanisation threshold can be estimated from the impervious-surface-area-ratio–bird-diversity-index graph, Threshold Indicator Taxa Analysis, or Locally Estimated Scatterplot Smoothing (LOESS). ‘LOESS’ is a non-parametric method involving locally weighted regression analyses, in which samples are divided into small intervals and polynomially fitted to each interval. The process is repeated to obtain weighted regression curves for each different interval, and the complete regression curve is synthesised at the centre of the individual regression curves [45]. Therefore, the ‘LOESS’ can help us calculate an urbanisation threshold when analysing the effect of urbanisation intensity on bird diversity. The response mode of river ecological indicators to urbanisation intensity can be roughly divided into three models: linear, buffer, and exponential. Of these, the exponential model is the best recognised.

Excel 2018 and Statistical Product and Service Solutions (SPSS v26.0. https://www.ibm.com/cn-zh/analytics/spss-statistics-software, accessed on 12 September 2021) were used to perform the alpha diversity analysis and statistical analysis. The RStudio (RStudio v1.4. https://www.rstudio.com/products/rstudio/, accessed on 21 September 2021) ‘Vegan’ and ‘ggplot2′ packages were used to perform the Pearson correlation analysis, RDA, and non-parametric ‘LOESS‘ regression analysis and to plot a stacked area chart.

## 3. Results

### 3.1. Bird Community Composition in the River Wetlands

In total, 124 species from 43 families and 13 orders, including 52 species of terrestrial birds and 72 species of waterbirds, were recorded during the 12-month survey period from 2020 to 2021 (Table S1). Fourteen species of threatened birds were observed, including the endangered Oriental Stork (*Ciconia boyciana*), the vulnerable Swan Goose (*Anser cygnoides*), Common Pochard (*Aythya ferina*), the near-endangered Northern Lapwing (*Vanellus vanellus*), Falcated Duck (*Anas falcata*), and Common Curlew (*Numenius arquata*), all of which were included in the Red List from the International Union for the Conservation of Nature (Table S1).

The bird species richness (H = 7.59, *p* > 0.05), Shannon–Wiener (H = 5.71, *p* > 0.05), and Pielou (H = 4.22, *p* > 0.05) indices of bird communities did not differ between individual study rivers in different seasons (Table 1 and Table S2).

Bird species richness differed between river sections (H = 11.84, *p* < 0.01; Table S3), being greater at sections of the lake entrance than at the middle sections (0.01 < *p* < 0.05; Table 2 and Table S4). Others were not significant (*p* > 0.05; Table 2, Tables S3 and S4).

The bird species richness (H = 30.29, *p* < 0.01), Shannon–Wiener (H = 38.66, *p* < 0.01), and Pielou (H = 45.81, *p* < 0.01) indices differed between seasons (Table 1, Table 2 and Table S5). The species richness index during winter was significantly greater than during spring (0.01 < *p* < 0.05) and summer (*p* < 0.01; Table S6). Moreover, the Shannon–Wiener (*p* < 0.01) and Pielou (*p* < 0.01) indices during winter was lower than that during autumn (Table S6). Others were not significant (*p* > 0.05; Tables S5 and S6).

### 3.2. Factors That Influence Bird Communities in River Wetlands

**Figure 2:**
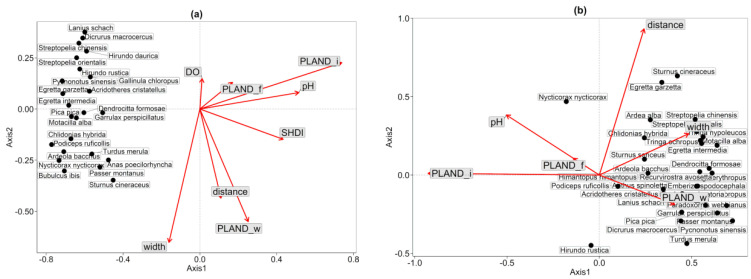

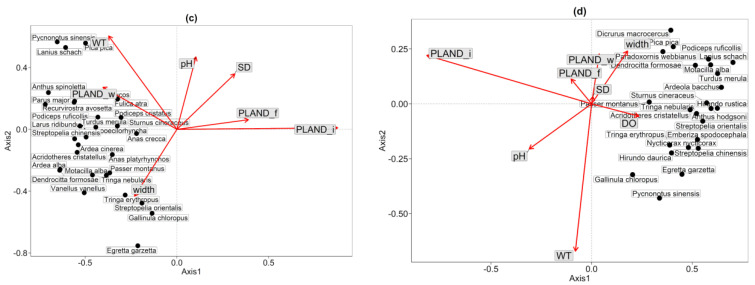
Relationship between bird communities and seasonal environmental factors. (**a**) Summer; (**b**) Autumn; (**c**) Winter; (**d**) Spring. pH (potential of hydrogen), DO (dissolved oxygen), WT (water temperature), SD (transparency), disturb (the degree of human disturbance), PLAND_i (the ratio of the impervious surface area), PLAND_w (the ratio of the water area), PLAND_f (the ratio of the forest area), SHDI (habitat heterogeneity), distance (the distance from the Hefei centre), and width (the width of each river section).

The sample points for bird species were well differentiated on both axis1 and axis2 in the RDA (Figure 2). Additionally, the PLAND_i has a significant impact on the diversity of bird communities in all four seasons.

The correlation analysis between axis1 and axis2 indicated that the environmental factor PLAND_i predominated during summer (*p* < 0.01), with a significant impact on bird community diversity (Figure 2a; Table 3). The correlation analysis between axis1 and axis2 indicated that the environmental factors PLAND_i (*p* < 0.01), distance (*p* < 0.01), and pH (*p* = 0.031) predominated during autumn, significantly impacting bird community diversity (Figure 2b; Table 3). The correlation analysis between axis1 and axis2 indicated that the environmental factor PLAND_i (*p* < 0.01) and WT (*p* < 0.01) predominated in winter, with a significant effect on bird community diversity (Figure 2c; Table 3). The correlation analysis between axis1 and axis2 indicated that the environmental factor PLAND_i predominated in spring (*p* = 0.018), impacting bird community diversity (Figure 2d; Table 3).

### 3.3. Influence of Urbanisation Intensity on Bird Communities in River Wetlands

#### 3.3.1. Trends in the Urbanisation Intensity of the Different River Sections

**Figure 3:**
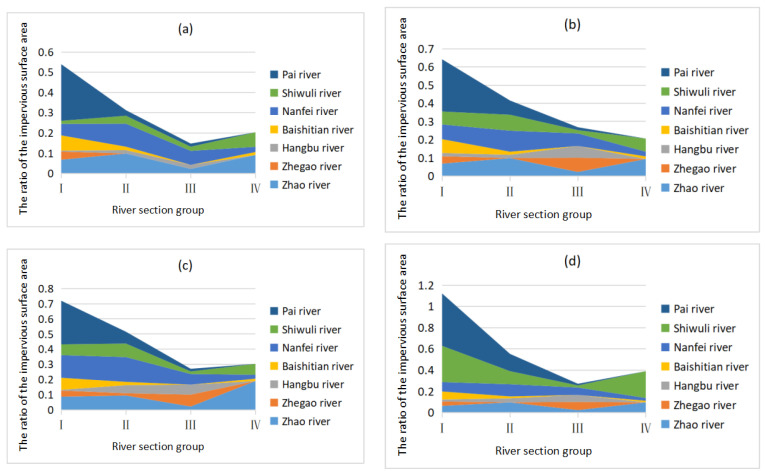
Trends in the urbanisation intensity of the different river section groups during each season. The coloured area denoting each river section in the figure represents the urbanisation intensity of that section. (**a**) Summer; (**b**) Autumn; (**c**) Winter; (**d**) Spring. I: river section group I; II: river section group II; III: river section group III; IV: river section group IV.

The urbanisation intensity of the rivers in the Chaohu Lake Basin decreased in section III, which was 5–10 km from the lake entrance (Figure 3). However, the three rivers in Hefei, especially the Nanfei River and Shiwuli River, had a high urbanisation intensity over their entire river basin, including the lake entrance.

#### 3.3.2. Distribution Pattern of Bird Communities along the Urbanisation Intensity Gradient

**Figure 4:**
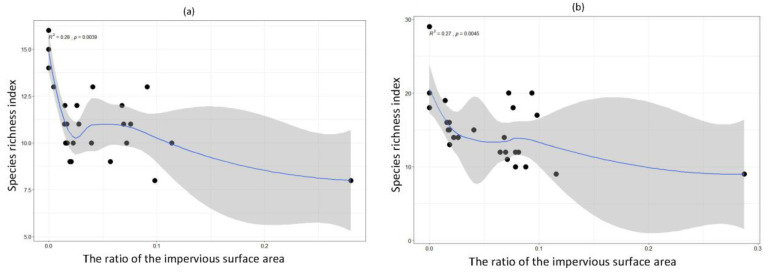

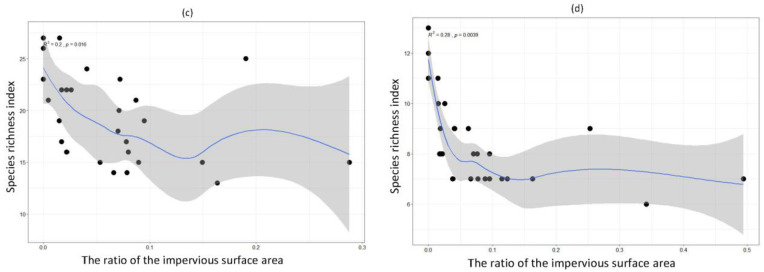
Non-linear regression between species richness index of waterbirds and urbanisation intensity. All data are log-transformed [*≥*(*x* + 1)]. The grey area indicates the confidential intervals for the fitted lines. (**a**) Summer; (**b**) Autumn; (**c**) Winter; (**d**) Spring. The dots in the figure represent the various river sections of the different rivers.

We selected the best-fitting species richness index to analyse the influence of urbanisation intensity on the diversity of both terrestrial bird and waterbird communities separately and found that the regression models describing the species richness index of terrestrial species were not significant (*p* > 0.05) in most seasons (Figure S2). In contrast, the explanatory power of the regression model applied to the species richness index of waterbirds was more significant (*p* < 0.05) in all four seasons, and the results fit the exponential decline model (Figure 4).

## 4. Discussion

The species richness at sections of the lake entrance was higher than that in the middle sections of the river, and the Shannon–Wiener index during winter was lower than that during autumn. The intensity of urbanisation had a significant impact on river birds in all seasons, and many other factors were coupled with the intensity of urbanisation. They also had a positive or negative impact on bird diversity in different seasons. Waterbird species richness indices had a negatively exponential correlation with urbanisation intensity and with the increase in urbanisation intensity; the rate of decline in species richness indices showed a trend of first being rapid and then becoming slower. However, terrestrial bird species richness indices had no significance with urbanisation intensity.

The lake entrance attracted more birds, predominantly Charadriiformes and Anseriformes, than other sections of the rivers for rest and foraging. The bird diversity in the Zhegao River and Pai River Wetlands that bordered the lake was higher than the other river sections. There were a variety of birds recorded that were on the IUCN Red List, such as Oriental Stork and Falcated Duck [13], suggesting that the entrance of the lake may be the original wetland environmental park providing suitable habitats for birds [4,7,22,37], which showed that the measures taken to establish biological reserves had achieved remarkable results in protecting rare birds and bird habitats in river wetlands. We also found that many threatened species only appeared in the less urbanised river sections during the investigation. Therefore, under rapid urbanisation, the fragmentation and loss of habitat caused by land development posed a great threat to sensitive groups and threatened species [46].

The intensity of human activity was an important factor in determining the biodiversity distribution pattern in urban ecosystems; therefore, anthropogenic factors had a much greater impact on biodiversity than natural environmental factors [2,20]. The distance from the urban centre indicated the pattern in which resources were distributed along the urbanisation gradient. Generally, the closer to the city centre, the higher the urbanisation [3,47,48]. We found that terrestrial birds and waterbirds appeared less frequently in more urbanised reaches during our survey. Following urbanisation, there were increases in the watershed population and the degree of artificial disturbance, which caused birds to invest more time in maintaining alert behaviour and led to reduced foraging time and energy intake, which ultimately affected their survival [20,39]. As the concentration and the total amount of pollutants in the rivers and lakes increased, the natural hydrological process changed in the basin [28,29]. In this case, bird species were extremely vulnerable to stress, and bird community diversity declined [35,37,49].

Urbanisation leads to an increase in the demand for construction, and the area covered by other land-use types (vegetation, grassland, water, etc.) was reduced or even lost. Canalisation, dams, and other hydraulic facilities reduced river habitat heterogeneity, forcing birds to choose nearby foraging grounds and habitats [1]. Environmental factors in the same habitat vary with season, and significant differences were observed in the structure of the bird communities at different times of a year [7,22,50]. It has previously been shown that impervious surface area factors were closely related to the distance from the urban centre, the degree of anthropogenic disturbance, and habitat heterogeneity [51,52], but the scope of this research was greater and took a longer time. Therefore, the intensity of urbanisation is an extremely important environmental factor for birds [44], as was confirmed in the RDA.

Our study statistically depicted the urbanisation gradient with the assistance of a valid land-use/land-cover (LULC) classification. Generally, we selected different habitat patches to represent the urbanisation gradient compared with the other urbanisation studies, in which the definition of ‘urbanisation intensity’ may be more statistically accurate [47,48]. The results of the RDA showed that the urbanisation intensity had a significant impact on river wetland birds in all four seasons [10,32]. This study also showed that community distribution patterns for terrestrial birds and waterbirds differed according to the gradient of urbanisation intensity, which were similar to those of Palacio et al. [53]. However, a more subtle variance in those terrestrial birds was not significantly affected by increased urbanisation. This may be because terrestrial birds rely less on river wetlands compared with waterbirds and because terrestrial birds are more adapted to urban environments [36]. The waterbird community diversity in river wetlands decreased exponentially with urbanisation increased [54]. The relationship between the diversity of waterbirds and the intensity of urbanisation was approximately linear when the urbanisation intensity was low, with a relatively fast decline rate at first, then a slower decline subsequently.

Our study has important implications for future urban landscape planning and the conservation of bird community diversity. The biological habitat available was insufficient when the ratio of impervious surfaces on both sides of the river wetland was high, which has a negative impact on bird diversity. The loss of habitat associated with urbanisation may be an important driver of the trends in bird diversity decline [5]. Therefore, it minimises the adverse effects of urbanisation by appropriate planning [47,55]. For example, river wetlands should be identified and protected as an important part of the urban ecosystem. Urban planners can reduce the extent of urbanisation in these areas by controlling the impervious surface area on both sides of the river and by reducing building area to expand bird habitat area [34,43,56]. Nevertheless, this study examines the correlation between different environmental factors and bird communities in river wetlands but does not comprehensively consider the impact of multiple factors on bird communities.

## 5. Conclusions

We explored the diversity of the bird community structure and its correlation with key environmental factors in different river sections. The results indicated that the species richness at sections of the lake entrance was higher than in the middle sections of the river. The urbanisation intensity was an important driving factor that led to changes in the structure of bird communities in river wetlands because of alterations in the land use types near river wetlands. Various habitat factors were highly coupled with urbanisation intensity, indicating a significant impact on birds in all four seasons. Our study confirmed that using the gradient in the distribution of landscape configurations of urban areas to study how land-cover changes affected bird diversity was ideal and that available habitats played a key role in determining the bird community structure along the urban intensity continuum. We also observed a trend indicating the negative impact of urbanisation on the abundance of bird species. We found that the rate of decline in species richness indices showed an initial rapid trend and subsequent slower changes with the increase in urbanisation intensity. Urbanisation has a significant impact on the abundance of waterbird species but not on terrestrial birds. The difference in the response of terrestrial birds and waterbirds to the intensity of urbanisation should receive more attention in future research on urbanisation gradients. The results of this study can help in planning future urban landscapes to protect the diversity of bird species by identifying and protecting river wetlands as an important part of the urban environment. Reducing the impervious surface along the river should be carried out as much as possible to protect and increase the area of bird habitat, especially at the lake entrance. Hence, the primary task for improving the effectiveness of biodiversity conservation is to reduce the intensity of urbanisation remains and to pay more attention to waterbird protection.

## Figures and Tables

**Table 1:** Diversity of birds observed in the different river wetlands during the different seasons.

Diversity	River	Summer	Autumn	Winter	Spring
Species richness index	Zhao River	34.33 ± 3.30	50.50 ± 3.50	51.80 ± 11.23	34.50 ± 0.50
	Zhegao River	39.33 ± 2.49	45.50 ± 0.50	46.20 ± 10.07	36.50 ± 2.50
	Hangbu River	34.00 ± 2.16	51.00 ± 3.00	39.80 ± 5.64	35.00 ± 3.00
	Baishitian River	36.00 ± 0.00	44.00 ± 2.00	45.40 ± 4.92	37.00 ± 5.00
	Nanfei River	33.67 ± 3.09	46.00 ± 6.00	34.80 ± 3.76	33.50 ± 0.50
	Shiwuli River	32.67 ± 3.68	48.50 ± 1.50	42.40 ± 5.85	34.50 ± 0.50
	Pai River	35.00 ± 2.94	40.50 ± 0.50	41.00 ± 8.44	35.50 ± 1.50
Shannon–Wiener index	Zhao River	3.00 ± 0.13	3.47 ± 0.06	2.71 ± 0.53	3.17 ± 0.01
	Zhegao River	3.01 ± 0.06	3.36 ± 0.01	2.80 ± 0.47	3.20 ± 0.07
	Hangbu River	2.72 ± 0.09	3.47 ± 0.01	2.53 ± 0.73	3.12 ± 0.02
	Baishitian River	3.01 ± 0.01	3.39 ± 0.04	2.79 ± 0.20	3.18 ± 0.05
	Nanfei River	2.81 ± 0.02	3.38 ± 0.09	2.41 ± 0.43	3.16 ± 0.02
	Shiwuli River	3.04 ± 0.05	3.45 ± 0.02	3.10 ± 0.26	3.21 ± 0.01
	Pai River	3.02 ± 0.07	3.32 ± 0.02	2.36 ± 0.67	3.15 ± 0.04
Pielou index	Zhao River	0.46 ± 0.03	0.56 ± 0.01	0.39 ± 0.08	0.50 ± 0.00
	Zhegao River	0.46 ± 0.01	0.55 ± 0.00	0.41 ± 0.09	0.52 ± 0.01
	Hangbu River	0.40 ± 0.02	0.57 ± 0.01	0.38 ± 0.13	0.50 ± 0.00
	Baishitian River	0.46 ± 0.00	0.57 ± 0.01	0.42 ± 0.03	0.50 ± 0.01
	Nanfei River	0.42 ± 0.00	0.57 ± 0.02	0.37 ± 0.08	0.50 ± 0.00
	Shiwuli River	0.46 ± 0.01	0.59 ± 0.00	0.50 ± 0.05	0.51 ± 0.00
	Pai River	0.46 ± 0.01	0.55 ± 0.00	0.33 ± 0.11	0.49 ± 0.01

**Table 2:** Diversity of birds in different river section groups during the four seasons. I: river section group I; II: river section group II; III: river section group III; IV: river section group IV.

Diversity	River Section Group	Summer	Autumn	Winter	Spring
Species richness index	I	45.00 ± 4.32	58.00 ± 2.00	53.20 ± 8.93	39.50 ± 6.50
	II	33.33 ± 0.94	57.50 ± 5.50	40.20 ± 8.91	31.50 ± 2.50
	III	36.33 ± 2.62	50.50 ± 1.50	44.20 ± 11.48	39.00 ± 1.00
	IV	47.33 ± 3.86	51.00 ± 1.00	62.20 ± 5.11	46.50 ± 2.50
Shannon–Wiener index	I	3.02 ± 0.07	3.52 ± 0.03	3.11 ± 0.36	3.25 ± 0.08
	II	2.87 ± 0.06	3.48 ± 0.07	3.03 ± 0.23	3.11 ± 0.01
	III	3.01 ± 0.07	3.41 ± 0.00	2.56 ± 0.47	3.15 ± 0.01
	IV	2.97 ± 0.04	3.45 ± 0.01	2.62 ± 0.69	3.27 ± 0.03
Pielou index	I	0.42 ± 0.01	0.54 ± 0.01	0.44 ± 0.06	0.49 ± 0.01
	II	0.41 ± 0.01	0.55 ± 0.01	0.45 ± 0.04	0.46 ± 0.00
	III	0.42 ± 0.02	0.51 ± 0.00	0.34 ± 0.07	0.45 ± 0.00
	IV	0.40 ± 0.00	0.51 ± 0.00	0.34 ± 0.10	0.46 ± 0.00

**Table 3:** The contribution rates of the two axes in the four seasons. a (Summer); b (Autumn); c (Winter); d (Spring).

Cumulative Contribution	a (Summer)	b (Autumn)	c (Winter)	d (Spring)
Axis1 (%)	77.30	64.77	51.86	65.29
Axis2 (%)	9.49	20.60	23.38	10.59
Cumulative contribution rate (%)	86.79	85.37	75.24	75.88

## Data Availability

The data presented in this study are available on request from the corresponding author.

## Supplementary Materials

The following supporting information can be downloaded at: https://www.mdpi.com/article/10.3390/ani12040473/s1, Table S1: The list of birds in seven river wetlands around Chaohu Lake, China; Table S2: Species richness, Shannon–Wiener, and Pielou indices among rivers using the Kruskal–Wallis test; Table S3: Species richness, Shannon–Wiener, and Pielou indices among river sections using the Kruskal–Wallis test; Table S4: Dunn–Bonferroni post hoc test between river section groups of species richness index; Table S5: Species richness, Shannon–Wiener, and Pielou indices among seasons using the Kruskal–Wallis test; Table S6: Dunn–Bonferroni post hoc test between the seasons of bird diversity; Figure S1: Pearson correlation matrix diagram of environmental factors in the river wetlands over the four seasons; Figure S2: Non-linear regression between terrestrial species richness and urbanisation intensity.
