# Supplementary material for: Impact of Urbanisation Intensity on Bird Diversity in River Wetlands around Chaohu Lake, China
Source: Animals (Basel). 2022 Feb 14;12(4):473. doi: 10.3390/ani12040473 (PMC8868527; doi:10.3390/ani12040473)
Supplement: Supplementary file 1 [file animals-12-00473-s001.zip › animals-1570166-supplementary.pdf]

Table S1. The list of birds in seven river wetlands around Chaohu Lake, China.

Common name[a]	Scientific name[a]	China[b]	IUCN[b]	River division[c]
	Galliforms			
Pheasants and Allies	Phasianidae			
1. Common Pheasant	*Phasianus colchicus*			A, B, C, D, E, F, G
	Anseriformes			
Ducks	Anatidae			
2. Swan Goose	*Anser cygnoides*	II	VU	A
3. Bean Goose	*Anser fabalis*		LC	A, D
4. Tundra Swan	*Cygnus columbianus*	II	LC	A
5. Ruddy Shelduck	*Tadorna ferruginea*			A, B, D, G
6. Common Shelduck	*Tadorna tadorna*			A, D
7. Northern Pintail	*Anas acuta*			C, D
8. Common Teal	*Anas crecca*			A, B, C, D, E, F, G
9. Falcated Duck	*Anas falcata*		NT	B, D, E, F, G
10. Mallard	*Anas platyrhynchos*			A, B, C, D, E, F, G
11. Spot-billed Duck	*Anas poecilorhyncha*			A, B, C, D, E, F, G
12. Gadwall	*Anas strepera*			B, F
13. Northern Shoveler	*Anas clypeata*			F, G
14. Common Pochard	*Aythya ferina*		VU	D
15. Common Merganser	*Mergus merganser*			A, C
	Podicipediformes			
Grebes	Podicipedidae			
16. Little Grebe	*Podiceps ruficollis*			A, B, C, D, E, F, G
17. Great-crested Grebe	*Podiceps cristatus*			A, B, C, D, E, F, G
	Columbiformes			
Pigeons	Columbidae			
18. Rufous Turtle Dove	*Streptopelia orientalis*			A, B, C, D, E, F, G
19. Red Turtle Dove	*Streptopelia tranquebarica*			B, C, E, F
20. Spotted Dove	*Streptopelia chinensis*			A, B, C, D, E, F, G
	Cuculiformes			
Cuckoos	Cuculidae			
21. Indian Cuckoo	*Cuculus micropterus*			D, G
22. Asian Koel	*Eudynamys scolopaceus*			B, C, D, E, F, G
23. Lesser Coucal	*Centropus bengalensis*	II		A, B, G
	Gruiformes			
Rails	Rallidae			
24. Brown Crake	*Amaurornis akool*			A, B, C, D, F, G
25. White-breasted Waterhen	*Amaurornis phoenicurus*			A, B, C, D, F
26. Common Moorhen	*Gallinula chloropus*		LC	A, B, C, D, E, F, G

27. Common Coot	*Fulica atra*		LC	A, B, C, D, E, F, G
	Charadriiformes			
Jacanas	Jacanidae			
28. Pheasant-tailed Jacana	*Hydrophasianus chirurgus*	II		A, B, D, E, F, G
Plovers	Charadriidae			
29. Northern Lapwing	*Vanellus vanellus*		NT	A, B, C, D, E, F, G
30. Grey-headed Lapwing	*Vanellus cinereus*			A, B, C, D, E, F, G
31. Kentish Plover	*Charadrius alexandrinus*			B, C, D, F
Sandpipers	Scolopacidae			
32. Eurasian Curlew	*Numenius arquata*	II	NT	A, B, C, D, G
33. Spotted Redshank	*Tringa erythropus*			A, B, C, D, E, F, G
34. Common Redshank	*Tringa totanus*			B, C, D, F, G
35. Common Greenshank	*Tringa nebularis*			A, B, C, D, E, F, G
36. Green Sandpiper	*Tringa ochropus*			A, B, C, D, E, F, G
37. Common Sandpiper	*Tringa hypoleucos*			A, B, C, D, E, F, G
38. Common Snipe	*Capella gallinago*			A, C, G
39. Eurasian Woodcock	*Scolopax rusticola*			G
Stilts	Recurvirostridea			
40. Black-winged Stilt	*Himantopus himantopus*			A, B, C, D, E, F, G
41. Pied Avocet	*Recurvirostra avosetta*			A, B, C, D, E, F, G
Gulls	Laridae			
42. European Herring Gull	*Laus argentatus*			A, B, C, D, E, F, G
43. Black-headed Gull	*Larus ridibundus*			A, B, C, D, E, F, G
44. Whiskered Tern	*Chlidonias hybrida*			A, B, C, D, E, F, G
	Ciconiiformes			
Herons	Ardeidae			
45. Grey Heron	*Ardea cinerea*			A, B, C, D, E, F, G
46. Chinese Pond Heron	*Ardeola bacchus*			A, B, C, D, E, F, G
47. Black-crowned Night-Heron	*Nycticorax nycticorax*			A, B, C, D, E, F, G
48. Eastern Great Egret	*Ardea alba*			A, B, C, D, E, F, G
49. Little Egret	*Egretta garzetta*			A, B, C, D, E, F, G
50. Intermediate Egret	*Egretta intermedia*			A, B, C, D, E, F, G
51. Eastern Cattle Egret	*Bubulcus ibis*			A, B, C, D, E, F, G
52. Yellow Bittern	*Ioxbrychus sinensis*			A, B, D
53. Black Bittern	*Ixobrychus flavicollis*			A, B, G
Storks	Ciconiidae			
54. Oriental White Stork	*Ciconia boyciana*	I	EN	B
Ibises	Threskiornithidae			
55. Eurasian Spoonbill	*Platalea leucorodia*	II		A, D, G
	Pelecaniformes			
Cormorants	Phalacrocorax			
56. Great Cormorant	*Phalacrocorax carbo*			C, E
	Bucerotiformes			

Hoopoes	Upupidae		
57. Eurasian Hoopoe	*Upupa epops*		A, B, C, D, E, F, G
	Coraciiformes		
Kingfishers	Alcedinidae		
58. Pied Kingfisher	*Ceryle rudis*		A, B, C, D, E, F, G
59. Common Kingfisher	*Alcedo atthis*		A, B, C, D, E, F, G
60. White-throated Kingfisher	*Halcyon smyrnensis*	II	A, B, C, D, F, G
	Piciformes		
Woodpeckers	Picidae		
61. Grey-headed Woodpecker	*Picus canus*		A, B, D, E, F, G
62. Great Spotted Woodpecker	*Dendrocopos major*		A, B, C, D, E, F, G
63. Grey-capped Pygmy Woodpecker	*Picoides canicapillus*		A, B, C, D, E, F, G
	Falconiformes		
Kites	Accipitridae		
64. Black Kite	*Milvus migrans*	II	A
65. Eastern Buzzard	*Buteo japonicas*	II	A
66. Upland Buzzard	*Buteo hemilasius*	II	C, D
Caracaras	Falconidae		
67. Common Kestrel	*Falco tinnunculus*	II	A, B
68. Eurasian Hobby	*Falco subbuteo*	II	B
	Passeriformes		
Larks	Alaudidae		
69. Oriental Skylark	*Alauda gulguta*		A, B, C, D, E, F, G
70. Eurasian Skylark	*Alauda arvensis*	II	A, B, C, D, F, G
Swallows	Hirundinidae		
71. Barn Swallow	*Hirundo rustica*		A, B, C, D, E, F, G
72. Red-rumped Swallow	*Hirundo daurica*		A, B, C, D, E, F, G
Wagtails	Motacillidae		
73. Eastern Yellow Wagtail	*Motacilla flava*		A, B, C, D, E, G
74. Grey Wagtail	*Motacilla cinerea*		A, B, C, D, E, F
75. White Wagtail	*Motacilla alba*		A, B, C, D, E, F, G
76. Olive-backed Pipit	*Anthus hodgsoni*		A, B, C, D, E, F, G
77. Water Pipit	*Anthus spinoletta*		A, B, C, D, E, F, G
78. Buff-bellied Pipit	*Anthus rubescens*		A, C, D, F
Bulbuls	Pycnontidae		
79. Light-vented Bulbul	*Pycnonotus sinensis*		A, B, C, D, E, F, G
80. Collared Finchbill	*Spizixos semitorques*		A, B, C, D, E, F, G
Thrushes	Turdidae		
81. Chinese Blackbird	*Turdus merula*		A, B, C, D, E, F, G
82. Dusky Thrush	*Turdus eunomus*		C, D, E, G
Cisticolas and Allies	Cisticolidae		
83. Zitting Cisticola	*Cisticola juncidis*		A, B, C, D, E, F, G
84. Plain Prinia	*Prinia inornata*		A, B, C, D, E, F, G

85. Siberian Stonechat	*Saxicola torquata*	A, B, C, D, E, F, G
Chats	Muscicapidae	
86. Red-flanked Bluetail	*Tarsiger cyanurus*	A, B, C, D, E, F, G
87. Oriental Magpie-Robin	*Copsychus saularis*	A, B, C, D, E, F, G
88. Daurian Redstart	*Pheonicurus auroreus*	A, B, C, D, E, F, G
Laughingthrushes Allies	Timallidae	
89. Masked Laughingthrush	*Garrulax perspicillatus*	A, B, C, D, E, F, G
Parrotbills and allies	Paradoxornithidae	
90. Vinous-throated Parrotbill	*Paradoxornis webbianus*	A, B, C, D, E, F, G
Cettia Bush Warblers and Allies	*Cettiidae*	
91. Japanese Bush Warbler	*Horornis diphone*	B, C, F, G
92. Dusky Warbler	*Phylloscopus fuscatus*	A, B, C, D, E
93. Yellow-browed Warbler	*Phylloscopus inornatus*	A, B, C, D, E, F, G
94. Pallas's Leaf Warbler	*Phyllosopus proregulus*	A, C, D, G
Bushtits	Aegithalidae	
95. Silver-throated Bushtit	*Aegithalos caudatus*	A, B, C, D, E, F, G
96. Black-throated Bushtit	*Aegithalos concinnus*	A, B, C, D, E, F, G
Tits	Paridae	
97. Yellow-bellied Tit	*Parus venustulus*	A, B, C, E, F, G
98. Great Tit	*Parus major*	A, B, C, D, E, F, G
White-eyes	Zosteropidae	
99. Swinhoe's White-eye	*Zosterops japonicus*	A, B, C, D, E, F, G
Figbirds	Oriolidae	
100. Black-naped Oriole	*Oriolus chinensis*	A, C, D, E, F, G
Shrikes	Laniidae	
101. Brown Shrike	*Lanius cristatus*	B, C, D, G
102. Long-tailed Shrike	*Lanius schach*	A, B, C, D, E, F, G
Drongos	Dicruridae	
103. Black Drongo	*Dicrurus macrocercus*	A, B, C, D, E, F, G
104. Ashy Drongo	*Dicrurus leucophaeus*	A, C, E, F, G
Starlings	Sturnidae	
105. White-cheeked Starling	*Sturnus cineraceus*	A, B, C, D, E, F, G
106. Black-collared Starling	*Gracupica nigricollis*	A, B, C, D, E, F, G
107. Red-billed Starling	*Sturnus sericeus*	A, B, C, D, E, F, G
108. Crested Myna	*Acridotheres cristatellus*	A, B, C, D, E, F, G
Jays	Corvidae	
109. Red-billed Blue Magpie	*Urocissa erythrorhyncha*	A, B, C, D, E, F, G
110. Azure-winged Magpie	*Dendrocitta formosae*	A, B, C, D, E, F, G
111. Oriental Magpie	*Pica pica*	A, B, C, D, E, F, G
112. Grey Treepie	*Dendrocitta formosae*	A, B, C, D, E, F, G
113. Daurian Jackdaw	*Corvus dauuricus*	A, B
114. Large-billed Crow	*Corvus macrorhynchos*	A, B
Old World Sparrows	Passeridae	
115. Eurasian Tree Sparrow	*Passer montanus*	A, B, C, D, E, F, G

Finches	Fringillidae	
116. Brambling	*Fringilla montifringilla*	A, B, D, F, G
117. Grey-capped Greenfinch	*Carduelis sinica*	A, B, C, D, E, F, G
118. Eurasian Siskin	*Carduelis spinus*	A, C, E, F, G
119. Chinese Grosbeak	*Eophona migratoria*	A, B, C, D, E, F, G
120. Japanese Grosbeak	*Eophona personata*	A, B, D
Waxbills	Estrildidae	
121. White-rumped Munia	*Lonchura striata*	A, B, C, D, E, F, G
Buntings	Emberizidae	
122. Yellow-throated Bunting	*Emberiza elegans*	A, B, D, F, G
123. Black-faced Bunting	*Emberiza spodocephala*	A, B, C, D, E, F, G
124. Little Bunting	*Emberiza pusilla*	A, B, C, D, E, F, G

Note: [a] The common and scientific names are based on Zheng (2017) (Zheng, G. A Checklist on the Classification and Distribution of the Birds of China, Third Edition; Science Press: Beijing, China, 2017.).

[b] Letters in parentheses indicate threatened birds listed in the International Union for Conservation of Nature (IUCN) Red List (www.iucnredlist.org): CR, Critically Endangered; EN, Endangered; VU, Vulnerable; NT, Near Threatened. I and II indicate the birds listed as national Grade I and Grade II key protected animals in China.

[c] The rivers entering Chaohu Lake (river division) are A (Zhao River), B (Zhegao River), C (Hangbu River), D (Baishitian River), E (Nanfei River), F (Shiwuli River), and G (Pai River).

Table S2. Species richness, Shannon–Wiener, and Pielou indices among rivers using the Kruskal–Wallis test.

Analysis item	Grouping variable	Sample size	Median	Standard deviation	Statistics	*p*-value
Species richness index	Zhao River	12	39.5	7.451	7.586	0.270
	Zhegao River	12	42	6.022		
	Hangbu River	12	37.5	7.416		
	Baishitian River	12	35	6.088		
	Nanfei River	12	38	6.613		
	Shiwuli River	12	41	11.819		
	Pai River	12	41	8.125		
	total	84	39	7.996		
Pielou index	Zhao River	12	0.504	0.055	5.707	0.457
	Zhegao River	12	0.461	0.059		
	Hangbu River	12	0.454	0.115		
	Baishitian River	12	0.418	0.095		
	Nanfei River	12	0.462	0.116		
	Shiwuli River	12	0.475	0.087		
	Pai River	12	0.481	0.081		
	total	84	0.472	0.09		
Shannon–Wiener index	Zhao River	12	3.152	0.229	4.222	0.647
	Zhegao River	12	3.003	0.268		
	Hangbu River	12	2.978	0.617		
	Baishitian River	12	2.822	0.491		
	Nanfei River	12	3.115	0.615		
	Shiwuli River	12	3.144	0.467		
	Pai River	12	3.098	0.388		
	total	84	3.083	0.462		

Note: The Kruskal–Wallis test results showed that the *p* values of the test results of the variables species richness, Shannon–Wiener, and Pielou indices were all > 0.05, so the statistical results were not significant, indicating that there was no significant difference in the species richness, Shannon–Wiener, and Pielou indices of different rivers.

Table S3. Species richness, Shannon–Wiener, and Pielou indices among river sections using the Kruskal–Wallis test. I: river section group I; II: river section group II; III: river section group III; IV: river section group IV.

Analysis item	Grouping variable	Sample size	Median	Standard deviation	Statistics	*p*-value
Species richness index	I	12	48.5	9.633	11.835	0.008**
	II	12	34	11.131		
	III	12	40	9.376		
	IV	12	51.5	8.475		
	total	48	47.5	10.97		
Shannon–Wiener index	I	12	3.142	0.301	1.94	0.585
	II	12	3.046	0.263		
	III	12	3.083	0.466		
	IV	12	3.122	0.574		
	total	48	3.103	0.421		
Pielou index	I	12	0.464	0.059	4.185	0.242
	II	12	0.457	0.053		
	III	12	0.427	0.082		
	IV	12	0.418	0.097		
	total	48	0.436	0.078		

Note: The asterisks ** represent the significance level 1%. The Kruskal–Wallis test results showed that, based on the species richness index, the *p* value of the test result was 0.008** < 0.05, indicating that there were significant differences among the different river sections.

Table S4. Dunn–Bonferroni post hoc test between river section groups of species richness index. I: river section group I; II: river section group II; III: river section group III; IV: river section group IV.

Two independent samples		Median		Adj *p*-value
Group item A	Group item B	Group item A	Group item B	
Richness_IV	Richness_I	51.5	48.5	1.000
Richness_IV	Richness_II	51.5	34	0.015*
Richness_IV	Richness_III	51.5	40	0.075
Richness_I	Richness_II	48.5	34	0.185
Richness_I	Richness_III	48.5	40	0.604
Richness_II	Richness_III	34	40	1.000

Note: The asterisk * represents the significance level 5%. Adj *p*-value means adjusted *p*-values by Dunn–Bonferroni post hoc test. Richness means species richness index.

Table S5. Species richness, Shannon–Wiener, and Pielou indices among seasons using the Kruskal–Wallis test.

Analysis item	Grouping variable	Sample size	Median	Standard deviation	Statistics	*p*-value
Species richness index	Summer	21	36	3.507	30.29	$P < 0.001$**
	Autumn	14	46.5	4.767		
	Winter	35	43	9.267		
	Spring	14	34.5	3.252		
	total	84	39	7.996		
Shannon–Wiener index	Summer	21	2.992	0.141	38.663	$P < 0.001$**
	Autumn	14	3.42	0.07		
	Winter	35	2.78	0.569		
	Spring	14	3.16	0.232		
	total	84	3.083	0.462		
Pielou index	Summer	21	0.459	0.029	45.808	$P < 0.001$**
	Autumn	14	0.564	0.016		
	Winter	35	0.434	0.102		
	Spring	14	0.499	0.041		
	total	84	0.472	0.09		

Note: The asterisks ** represent the significance level 1%. The Kruskal–Wallis test results showed that the *p*-values of the test results of the variables about species richness, Shannon–Wiener, and Pielou indices were all < 0.05, indicating that there was significant difference in the species richness, Shannon–Wiener, and Pielou indices of different seasons.

Table S6. Dunn–Bonferroni post hoc test between the seasons of bird diversity.

Two independent samples		Median		Adj *p*-value
Group item A	Group item B	Group item A	Group item B	
Pielou_Summer	Pielou_Autumn	0.459	0.564	*P* < 0.001**
Pielou_Summer	Pielou_Winter	0.459	0.434	1.000
Pielou_Summer	Pielou_Spring	0.459	0.499	0.024*
Pielou_Autumn	Pielou_Winter	0.564	0.434	*P* < 0.001**
Pielou_Autumn	Pielou_Spring	0.564	0.499	0.163
Pielou_Winter	Pielou_Spring	0.434	0.499	0.003**
S-W_Summer	S-W_Autumn	2.992	3.42	*P* < 0.001**
S-W_Summer	S-W_Winter	2.992	2.78	1.000
S-W_Summer	S-W_Spring	2.992	3.16	0.139
S-W_Autumn	S-W_Winter	3.42	2.78	*P* < 0.001**
S-W_Autumn	S-W_Spring	3.42	3.16	0.046*
S-W_Winter	S-W_Spring	2.78	3.16	0.066
Richness_Summer	Richness_Autumn	36	46.5	*P* < 0.001**
Richness_Summer	Richness_Winter	36	43	0.002**
Richness_Summer	Richness_Spring	36	34.5	1.000
Richness_Autumn	Richness_Winter	46.5	43	0.258
Richness_Autumn	Richness_Spring	46.5	34.5	*P* < 0.001**
Richness_Winter	Richness_Spring	43	34.5	0.026*

Note: The asterisks ** and * represent the significance levels 1% and 5%, respectively. Richness, S-W, Pielou means species richness, Shannon–Wiener and Pielou indices. Adj *p*-value means adjusted *p*-values by Dunn–Bonferroni post hoc test.

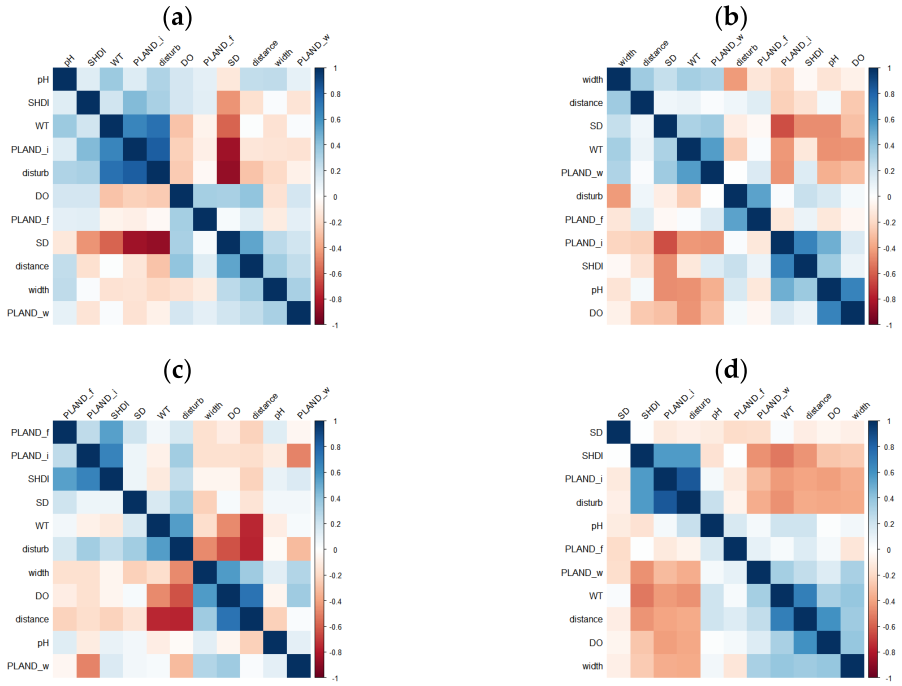

Figure S1. Pearson correlation matrix diagram of environmental factors in the river wetlands over the four seasons. A deeper red colour indicates a stronger positive correlation, whereas a deeper blue colour indicates a stronger negative correlation. a: Summer; b: Autumn; c: Winter; d: Spring.

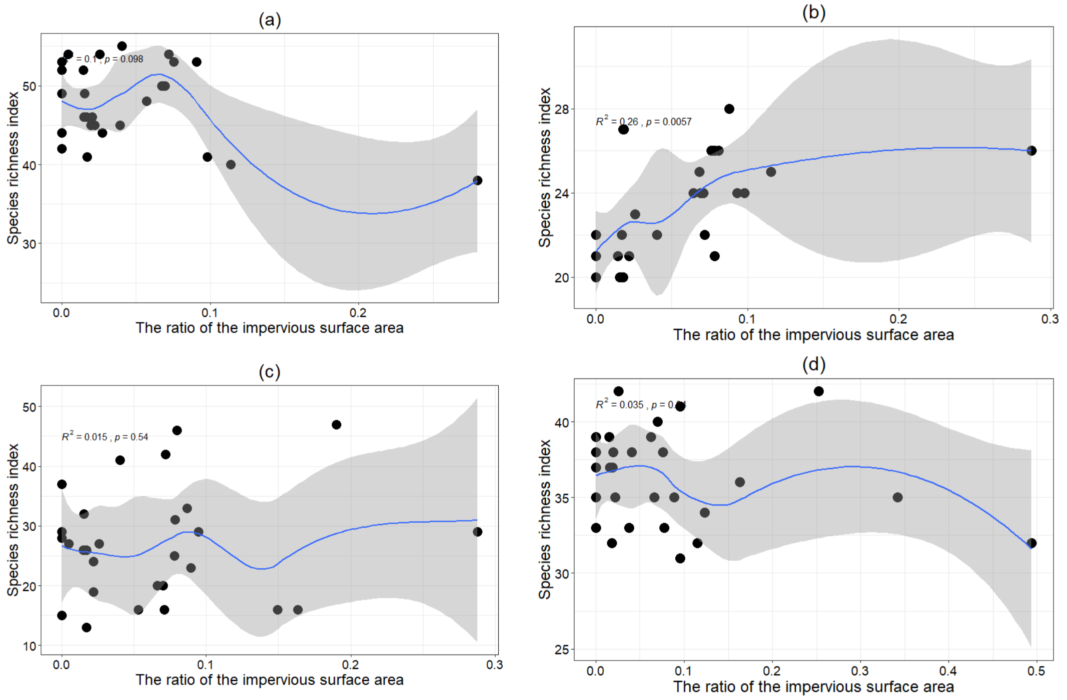

Figure S2. Non-linear regression between terrestrial species richness and urbanisation intensity. All data were log-transformed [≥ (x + 1)]. The grey area indicates the confidential intervals for the fitted lines. a: Summer; b: Autumn; c: Winter; d: Spring.
